# Emotional Intelligence and Academic Self-Efficacy in Relation to the Psychological Well-Being of University Students During COVID-19 in Venezuela

**DOI:** 10.3389/fpsyg.2021.759701

**Published:** 2021-12-15

**Authors:** Diego García-Álvarez, Juan Hernández-Lalinde, Rubia Cobo-Rendón

**Affiliations:** ^1^Departamento de Ciencias del Comportamiento, Universidad Metropolitana, Caracas, Venezuela; ^2^Facultad de Ciencias Básicas y Biomédicas, Universidad Simón Bolívar, Cúcuta, Colombia; ^3^Laboratorio de Investigación e Innovación Educativa (IDECLAB), Dirección de Docencia, Universidad de Concepción, Concepción, Chile

**Keywords:** COVID-19, higher education, emotional intelligence, academic self-efficacy, psychological well-being

## Abstract

Due to the COVID-19 pandemic, educational centers and universities in Venezuela have closed their physical plants and are migrating to emergency remote education to continue with academic programs. This empirical study aimed to analyze the predictive capacity of academic self-efficacy and emotional intelligence skills on each of the dimensions of psychological well-being. We employed a cross-sectional predictive design. The sample comprised 277 university students, of which 252 were female (91.00%). Their ages ranged from 18 to 45 years, with a mean of 20.35 (*SD* = 2.29). Non-probabilistic chance sampling was used. For data collection, we used an anonymous online form, contacted students by mail, and invited them to participate in the study. Questionnaires were available between 217 and 227 days of decreed quarantine in Venezuela. The results indicated average levels of academic self-efficacy (Me = 4; IQR = 2), emotional intelligence: clarity (Me = 27; IQR = 10), attention (Me = 25; IQR = 10) y repair (Me = 25; IQR = 12), and psychological well-being (Me = 35; IQR = 5). We found differences according to sex and age, specifically in emotional regulation (*z* = 3.73, *p* < 0.001, *d* = 0.438) and in bonds of psychological well-being (*z* = 2.51, *p* = 0.012, *d* = 0.276) favoring men (Me = 33, IQR = 9; Me = 8, IQR = 1), respectively. Regarding age, statistically significant differences were found in the group of students older than 21 years with higher perception of psychological well-being (*z* = 3.69, *p* < 0.001, *d* = 0.43) and in each of its dimensions. Emotional intelligence and academic self-efficacy were found to be significant predictors of psychological well-being and its dimensions, specifically on control (*R*^2^-Cox = 0.25, *R*^2^-Nagelkerke = 0.34, 69.90% of total correct classification), links (*R*^2^-Cox = 0.09, *R*^2^-Nagelkerke = 0.12, 65.07% of total correct classification), projects (*R*^2^-Cox = 0.32, *R*^2^-Nagelkerke = 0.46, 78.40% of total correct classification), acceptance (*R*^2^-Cox = 0.17, *R*^2^-Nagelkerke = 0.23, 68.28% of total correct classification), and total well-being (*R*^2^-Cox = 0.52, *R*^2^-Nagelkerke = 0.71, 87.16% of total correct classification). It was concluded that emotional intelligence and academic self-efficacy are protective psychological resources of psychological well-being that should be promoted at university to mitigate the negative effects of the pandemic on the mental health of young people.

## Introduction

In Venezuela, since March 2020, the authorities have taken preventive measures to counteract the spread of the new coronavirus (SARS-Cov-2); these have included quarantine, social distancing, isolation, and even confinement (García-Álvarez and Cobo-Rendón, 2020). One of the consequences of these measures was the drastic shift from face-to-face university education to a model of university education called university at home. In short, it was a transition from dynamic face-to-face classes to distance learning, in a social fabric of uncertainty and high expectations. This required a constant reconstruction of the formal educational training processes in a country where there are already so many technological obstacles. Studies have reported student desertion, discouragement, and lack of support regarding motivating to participate, as a possible consequence of this implementation of emergency remote education in Latin America (García-Aretio, 2021).

Recent emerging scientific literature on student psychological well-being during COVID-19 has shown, *via* empirical studies, that students’ psychological well-being is low (Dodd et al., 2021) with impairments in mental and physical health (Son et al., 2020). Other longitudinal studies have also shown that students’ psychological well-being and mental health have decreased (Savage et al., 2020). In Saudi Arabia, university students reported a high prevalence of anxiety, insomnia, and depression during confinement in 2020, and family support was found to be a protective factor of well-being, especially in women (Alfawaz et al., 2021). In the United Kingdom, it was found that more than half of the student population had mental health difficulties, such as stress, anxiety, depression, and even symptoms of psychological trauma. At the same time, it was found that self-efficacy and physical exercise appeared to be coping mechanisms during confinement (Ihm et al., 2021).

Similarly, in Sweden, university students reported negative mental health experiences, such as loneliness, boredom, anxiety, stress, depressive symptoms, and concerns regarding their studies, finances, and possible contagion. In addition, they reported high-stress negative affects such as interruption of studies, confinement, and uncertainty due to the pandemic, which impacted academic self-efficacy (Berman et al., 2021). In Spain too, students reported increased anxiety during their 2020 confinement. It was also found that students with higher levels of anxiety had lower levels of academic self-efficacy, specifically male students reported higher levels of self-efficacy that the women (Alemany-Arrebola et al., 2020).

Studies have focused on personal resources as well as those related to academic contexts. University students have other outlets that bring them gratification, showing that despite adversities and difficulties, university students have found spaces of gratification in the university context, even suggesting that self-efficacy, academic satisfaction, and sense of belonging are configured as protective factors (Capone et al., 2020). Self-efficacy can be a protective factor against student insecurity and stress during this period (Gulley et al., 2021; Wen et al., 2021). It has been identified as a predictor of academic performance during COVID-19 pandemic (Talsma et al., 2021). A study in China found a positive relationship between academic self-efficacy and student well-being (Yang et al., 2021). In addition, in September 2020, another study found that university students reported good psychological well-being when some of the preventive measures were lifted by April 2020, such as the return to classes at some universities (Tan et al., 2021). Emotional self-efficacy has also been found to be negatively associated with fear of contagion in Italian university students. Low levels of emotional and academic self-efficacy have been associated with depressive symptoms and learning skill deficits during confinement (Calandri et al., 2021).

Another personal resource that has been studied during the pandemic is emotional intelligence. Emotional intelligence is a protective factor of well-being (Hussien et al., 2020; Bermejo-Martins et al., 2021; Moroń and Biolik-Moroń, 2021), and directly and indirectly contributes to improving psychoeducational variables in university students (Iqbal et al., 2021). It has aided in the development of resilience, well-being, and mental health in Spanish students during the COVID-19 pandemic (Bartos et al., 2021). Specifically, emotional intelligence is a predictor of psychological well-being, enabling healthier coping strategies during COVID-19 pandemic by avoiding emotional distress (Sánchez-Ruiz et al., 2021). Some experimental studies based on emotional intelligence and mindfulness in university students report a decrease in anxiety and depression symptoms during the confinement period (Sturgill et al., 2021). Another intervention based on the improvement of emotional intelligence through workshops and seminars for university students improved the levels of academic performance, academic engagement, and self-efficacy during confinement. The authors of this intervention suggest the importance of including training in socioemotional education in the university curriculum (Moreno-Fernández et al., 2020); other studies have confirmed that emotional intelligence has an impact on burnout, stress, and academic performance in the development of e-learning confined (Alam et al., 2021). It is relevant to consider emotional skills and academic self-efficacy as psychoeducational variables that may impact the adequate psychosocial functioning of students, that is, with self-perceived psychological well-being as an indicator of mental health (Ryff et al., 2021).

We would like to point out some important concepts of the variables that underpin the research we are presenting here:

a) The emotional intelligence construct formulated by Salovey and Mayer (1990) refers to a cognitive capacity for attention, understanding, and regulation of emotions—all skills that allow for the development of a healthier life (Salovey et al., 1995).
b) Academic self-efficacy, a construct of Bandura’s (1982) social cognitive theory, is defined as the beliefs and expectations that people have regarding their ability to plan, organize, and execute specific activities of academic performance (Palenzuela, 1983; Domínguez-Lara and Merino-Soto, 2017).
c) Multidimensional psychological well-being (Ryff, 1989), a concept that was further developed in South America by Casullo (2002) is understood as a broad construct that includes social, subjective, and psychological dimensions that lead people to function well. It is composed of six dimensions: (c1) Self-acceptance, which is also a main characteristic of positive functioning. People with high self-acceptance have a positive attitude toward themselves, feel good about their past, and accept the different aspects of their personality, both positive and negative; (c2) Positive relationships with other people. This refers to the capacity to experience love, empathy, and intimacy with others and to have quality relationships with others; (c3) Autonomy, which implies the capacity to maintain one’s individuality in various social contexts, such as resisting social pressure and self-regulation of behavior, enjoying self-determination, and maintaining independence and personal authority; (c4) Mastery of the environment, which refers to managing the demands and opportunities of the environment to satisfy one’s own needs and abilities, as well as the capacity to influence the context to which one belongs; (c5) Purpose in life, that is, the capacity to set goals and define a series of objectives that allow one’s life to have meaning and purpose; and (c6) Personal growth, which encompasses the interest in developing potential, growing as a person, and maximizing one’s own potential.

In Latin America, we found some antecedents focused on psychological well-being in university students during the COVID-19 pandemic. In Peru, researchers found a deterioration of psychological well-being relating to low psychological coping strategies (Campos et al., 2020). In another study in Guatemala, with a higher percentage of female participation (66.7%), the authors also found average levels of multidimensional psychological well-being during COVID-19 confinement. Possibly explained by resilient factors and low anxiety (González-Aguilar, 2021).

In Colombia, with a sample of Psychology students that included a greater number of female participants (80.4%), they found average levels of multidimensional psychological well-being, although with low dimensions of social relationships or bonds, as well as in the dimension of autonomy, measured during weeks six and nine of declared preventive social isolation in the country (Araque-Castellanos et al., 2020). In the same context, another study was developed on psychological well-being in Psychology students. A greater female participation was identified (78.85%). The authors found high levels of multidimensional psychological well-being, suggesting that there were possibly socio-cultural or psychological factors that could protect the mental health of students (Ruiz-Domínguez et al., 2020). It is interesting to note that, in several studies on the effects of the pandemic on student well-being, women presented greater participation in these investigations.

The COVID-19 pandemic is considered a healthcare crisis. In Venezuela, this is combined with political, social, and economic crises. Against this background, this study seeks to generate paths of action educational and intervention to prevent psychosocial risks and promote mental health of undergraduates. Some studies conducted in 2020 have shown high levels of anxiety, emotional intelligence (Giménez and Medici, 2021), increased stress, and depression without risk of psychosis in the Venezuelan general population (Raggio et al., 2021). A study with university students in Maracaibo, Venezuela reported that students maintained an adequate level of self-efficacy that allowed them to manage their academic load, despite difficulties in planning and completion of tasks in a timely manner (Romero et al., 2021). The antecedents at the global, Latin American, and national levels suggest the relevance of studying psychological well-being as a relational variable in the educational context during the pandemic.

This study is part of the research trends referred to in the “university student” person. This research seeks to understand psycho-social factors that influence mental health and university experience in a period of life considered highly stressful due to the different demands and conditions of adaptation, permanence, and progress in university studies. In the same way, there is a growing interest in psychological and educational science in studying personal and social resources that can be considered as protective or resilient factors in times of pandemic, an event that can be considered psychosocial trauma. We are interested in studying the personal and social resources that can be considered as protective and that can mitigate the negative effects of the pandemic on students at the university level (UNESCO, 2020; Parra-Sandoval, 2021).

Therefore, this research is justified in contributing to the study of university students in a person-oriented approach, emphasizing psychological well-being in relation to emotional intelligence skills, including a psychoeducational variable such as academic self-efficacy in the research design. Study carried out during a COVID-19 pandemic in a country with a generalized social, economic, and political crisis. The contributions of this study are: (a) approach to the object of well-being from a salutogenic approach, and not from an approach based on the detection or screen of symptoms; (b) corroborate that personal factors such as emotional intelligence and academic self-efficacy can be protective factors of student psychological well-being in crisis situations such as the pandemic; (c) the results can contribute to the design of psychosocial interventions for students that can enhance personal resources as resilient factors, it is also important to take into account the gender approach for the construction of well-being; and (d) In a Latin American context, the manuscript stands out as an important background in studying the variables mentioned in a pandemic context that gives a cross-sectional impression of how students have maintained their well-being. Studies are needed to explore mental health, the impact of educational changes on psychological well-being, as well as health promotion in the development of the pandemic, but also in the post-pandemic return to school (Wang et al., 2021).

The objectives of the research were: (a) to describe emotional intelligence skills, academic self-efficacy and multidimensional psychological well-being in university students in Venezuela during the COVID-19 pandemic; (b) to determine whether there are statistically significant differences according to gender and age of the students; and (c) to analyze the predictive capacity of academic self-efficacy and each of the emotional intelligence skills on each of the dimensions of psychological well-being.

## Materials and Methods

This is a cross-sectional and empirical-predictive study, with non experimental research design. It is a cross-sectional study, since only one measurement of the variables of interest we are made. It is of an empirical-predictive type, since the analysis of the variables we are carried out employing quantitative methods (Ato et al., 2013).

### Participants

The sample consisted of 277 students from a Venezuelan university, of which 252 were female (91.00%). Their ages ranged from 18 to 45 years, with a mean of 20.35 (*SD* = 2.29). When grouped by age, a percentage of 58.80% (*n* = 163) was recorded for those under 20 years of age, and 41.2% (*n* = 114) for those aged 21 years and older. The difference in sex can be explained by the fact that the Psychology program has more female enrollment. The sample was obtained employing a non-probabilistic sampling of casual or accidental type according to Kerlinger and Lee (2002) in this research it was specifically casual by volunteers who decided to participate in the call for the study.

### Instruments

A questionnaire was made available online between 217 and 227 days of the decreed quarantine in Venezuela (October 20 to 30, 2020). The form contained informed consent, the objective of the study, and the following psychometric scales:

a) Multidimensional Psychological Well-being Scale: its objective is to measure psychological well-being from the eudemonic perspective. It is a 13-item scale with three response options: disagree (1), neither agree nor disagree (2), and agree (3). The confirmatory factor analyses in the original study yielded four dimensions of psychological well-being.
The dimensions are Control/Acceptance (e.g., “If something goes wrong I can accept it, admit it”), Autonomy (e.g., “I can say what I think without major problems”), bonds (e.g., “I have people to help me if I need it”), and Projects (e.g., “I think I know what I want to do with my life”) in addition to adequate reliability (Casullo, 2002). For the Venezuelan context, it presents adequate psychometric indices (García-Álvarez and Hernández-Lalinde, 2020).
b) Trait Meta-Mood Scale (TMMS) with the aim of measuring emotional intelligence from the model of Salovey and Mayer (1990), is a scale designed in Spanish with 24 items that has five response options: do not agree at all (1), somewhat agree (2), quite agree (3), strongly agree (4), and strongly agree (5), for its correction the scores in each of the three dimensions are added directly. The dimensions are perception (e.g., “I think it is worth paying attention to my emotions and mood”), understanding (e.g., “I often notice my feelings in different situations”), and emotional regulation (e.g., “even if I feel bad, I try to think about pleasant things”). This questionnaire has adequate psychometric indices (Fernández-Berrocal et al., 2004).
c) A single item measuring academic self-efficacy- “How confident are you that you will be able to effectively perform the tasks (papers, exhibits, exams, etc.) that your academic life demands of you?” (Domínguez-Lara and Merino-Soto, 2017). It is answered with a five-point scale ranging from “not at all confident” (1) to “very confident” (5). Previous studies in South America have shown that this unique item of academic self-efficacy that has adequate validity evidence with other personality constructs and other self-efficacy measures (Domínguez-Lara et al., 2019).

Table 1 lists the reliability coefficients of the questionnaires used. The selected questionnaires presented adequate validity and reliability indices for their use among university students.

**TABLE 1:** Participants’ level of academic self-efficacy, emotional intelligence, and psychological well-being.

Variable	Me (IQR)	Cronbach’s alpha	McDonald’s omega
Academic self-efficacy	4 (2)	–	–
EI–Attention	27 (10)	0.90	0.90
EI–Clarity	25 (10)	0.89	0.89
EI–Repair	25 (12)	0.89	0.89
PB–Control	11 (2)	0.61	0.62
PB–Links	9 (1)	0.62	0.63
PB–Projects	8 (1)	0.45	0.46
PB–Acceptance	8 (2)	0.48	0.48
PB–Total	35 (5)	0.82	0.82

*EI, emotional intelligence; PB, psychological well-being; Me, median; IQR, interquartile range.*

### Procedure

Data collection was performed using an online form. The form also mentioned the objective of the study and emphasized anonymity, confidentiality, and the scientific nature of the results, before requesting for consent to participate in the study. This study was conducted in accordance with the ethical guidelines of the American Psychological Association, Declaration of Helsinki, and the Federation of Psychologists of Venezuela.

### Analysis Plan

The normal distribution of scores was verified using the Shapiro-Wilk test, box plots, and Q-Q plots. We did not find normal distribution of scores, the Mann-Whitney *U* test was used to compare the constructs analyzed according to sex and grouped age. To express the results, the median (Me) and the interquartile range (IQR) were used, and the mean rank (MR) was adopted as a measure of centralization in cases in which the distribution of scores exhibited a different shape between groups. The predictive role of emotional intelligence and academic self-efficacy was established through logistic regression, in which the dependent variables were each of the dimensions of psychological well-being. These were categorized using the 25th and 75th percentiles, thus creating two groups identified as “low” and “high” levels of psychological well-being. In addition, age and sex were included as covariates, and adjusted odds ratios were obtained. The assumptions of the logistic regression were verified without finding inconsistencies, while the predictive capacity and model fit were established using the classification table and the Cox and Nagelkerke pseudo coefficients of determination. The data were processed and statistically analyzed in IBM SPSS Statistics 25 and R version 4.0.5.

## Results

The results showed low average levels of emotional intelligence skills in the following order: comprehension (Me = 27; IQR = 10), attention (Me = 25; IQR = 10), and regulation (Me = 25; IQR = 12). Regarding self-perceived academic self-efficacy, students reflected an adequate level of confidence in their competence to efficiently fulfill their academic duties during the pandemic (Me = 4; IQR = 2). Results showed average levels of psychological well-being (Me = 35; IQR = 5), suggesting the need for psychological intervention to promote adequate psychosocial functioning in the dimensions of control, self-acceptance, autonomy, bonds, and Project or purpose in life. See Table 1 for descriptive analyses of variables and reliability indicators.

### Differentiation According to Sociodemographic Characteristics

Gender had an effect only on emotional regulation, a dimension corresponding to emotional intelligence, and on bonding, a construct pertaining to psychological well-being. Specifically, male students (Me = 33, IQR = 9) exhibited higher scores than female students (Me = 25, IQR = 12.75) in emotional regulation or repair, we detected a significant difference of small magnitude (*z* = 3.73, *p* < 0.001, *d* = 0.44). Likewise, the bonds reported by male students (MR = 190.31, IQR = 1) were greater than those found in female students (MR = 148.25, IQR = 1), again showing a small effect size (*z* = 2.51, *p* = 0.012, *d* = 0.28). In contrast, age influenced all dimensions of psychological well-being, including the total construct; participants aged 21 years or older had higher levels of psychological well-being (see Table 2).

**TABLE 2:** Psychological well-being according to age groups.

Variable	Up to 20 years: MR (IQR)	From 21 years: MR (IQR)	*Z* (p)	Cohen’s d
Control	143.70 (2)	165.77 (2)	2.20 (0.028)	0.25
Links	143.82 (2)	165.56 (1)	2.31 (0.021)	0.24
Projects	143.61 (1)	165.90 (1)	2.26 (0.024)	0.25
Acceptance	141.15 (1)	169.98 (2)	2.89 (0.004)	0.32
Psychological well-being	137.67 (5)	175.76 (3.25)	3.69 (<0.001)	0.43

*MR, mean rank; IQR, interquartile range. Mean rank are reported instead of median because the shape of the distributions differs according to age group.*

### Predicting Psychological Well-Being Based on Emotional Intelligence and Academic Self-Efficacy

Our findings regarding the predictive role of emotional intelligence and academic self-efficacy on psychological well-being are shown in Table 3. The results showed that emotional attention decreases high scores in psychological well-being and its dimensions: acceptance, bonds, and projects; emotional understanding increases scores in psychological well-being and its dimensions of acceptance, autonomy, and projects; and emotional regulation increases scores in the dimension of bonds. In turn, academic self-efficacy predicts higher scores in psychological well-being, acceptance, and bonding dimensions. Taking the acceptance/control dimension as an example, we observed that a one-point increase in emotional attention decreases the probability of obtaining a high level in this construct by 6.20% (OR = 0.94, 95% CI: 0.89–0.99), while unit increases in the emotional clarity dimension raise the probability of achieving high scores on the dimension by 7.45% (OR = 1.07, 95% CI: 1.01–1.15). In contrast, a one-point increase in academic self-efficacy raises the probability of exhibiting a high level in this dimension by 163.39% (OR = 2.63, 95% CI: 1.78–3.90). It should be clarified that the factors not described in these results were assumed to have remained constant during the study period. In addition, both age and sex were included as covariates in the regression models.

**TABLE 3:** Emotional intelligence and academic self-efficacy as predictors of psychological well-being.

Dependent variable (DV)	Independent variables (IV)	OR (p)	CI 95%	*R* ^2^	Sens (%)	Spec (%)	T (%)
Control	Emotional attention	0.94 (0.022)	0.89–0.99	0.25, 0.34	71.17	68.42	69.90
	Emotional clarity	1.07 (0.040)	1.00–1.15				
	Emotional repair	1.02 (0.474)	0.97–1.08				
	Academic self-efficacy	2.63 (<0.001)	1.78–3.90				
	Age	1.17 (0.065)	0.99–1.38				
	Gender	0.47 (0.246)	0.13–1.68				
Links	Emotional attention	0.97 (0.183)	0.93–1.01	0.09, 0.12	53.23	75.00	65.07
	Emotional clarity	1.09 (0.002)	1.03–1.15				
	Emotional repair	0.99 (0.636)	0.95–1.03				
	Academic self-efficacy	1.29 (0.070)	0.98–1.69				
	Age	1.12 (0.086)	0.98–1.27				
	Gender	0.83 (0.699)	0.33–2.10				
Projects	Emotional attention	0.85 (<0.001)	0.79–0.91	0.32, 0.46	50.70	89.39	78.40
	Emotional clarity	1.04 (0.256)	0.97–1.12				
	Emotional repair	1.08 (0.012)	1.02–1.15				
	Academic self-efficacy	3.35 (<0.001)	2.17–5.19				
	Age	1.54 (<0.001)	1.22–1.95				
	Gender	1.16 (0.831)	0.31–4.35				
Acceptance	Emotional attention	0.90 (<0.001)	0.85–0.95	0.17, 0.23	73.53	61.90	68.28
	Emotional clarity	1.13 (0.001)	1.05–1.21				
	Emotional repair	1.01 (0.750)	0.95–1.07				
	Academic self-efficacy	1.18 (0.362)	0.83–1.68				
	Age	1.15 (0.061)	0.99–1.33				
	Gender	0.41 (0.189)	0.11–1.55				
Total	Emotional attention	0.68 (<0.001)	0.58–0.80	0.52, 0.71	83.61	89.66	87.16
	Emotional clarity	1.46 (<0.001)	1.23–1.74				
	Emotional repair	1.10 (0.078)	0.99–1.22				
	Academic self-efficacy	2.81 (0.001)	1.56–5.04				
	Age	0.96 (0.964)	0.17–5.43				
	Gender	1.36 (0.081)	0.96–1.92				

*OR, odds ratio; CI, bilateral confidence interval; R^2^, Cox and Nagelkerke pseudo R^2^; Sens, sensitivity; Spec, specificity; T, total.*

## Discussion

Our central objective was to determine the predictive role of academic self-efficacy and emotional intelligence skills on the psychological well-being of university students during a period of confinement due to the COVID-19 pandemic. Despite the difficulties related to the pandemic, university students show an average level of psychological well-being, possibly promoted by their personal resources of emotional understanding and regulation. We also found an adequate level of academic self-efficacy, possibly allowing them to organize motivational and behavioral efforts to perform tasks, reports, exams, and other activities demanded by university academic life.

Regarding emotional intelligence skills (Fernández-Berrocal et al., 2004), the participating students showed adequate attention. Students report the ability to read their emotions and feelings by being able to experience them. They can consciously recognize their emotions and identify what they feel by giving it a verbal label. At the level of emotional understanding, the participating students can understand themselves, identifying their needs and desires, being able to integrate what they feel within their complex thinking considering the changes according to the context marked by adversity.

The university students presented adequate emotional regulation. Emotional regulation is a skill that would help them to control their own emotional response to intense situations. It is suggested that students can generate alternative adaptive thoughts to control/regulate the different variations that may occur during the day. Can tolerate frustration and feel confident in the face of goals they set in the midst of a pandemic. This finding is congruent with their level of academic self-efficacy, which was adequate. Possibly helping them to continue in their training process despite difficulties, suggesting that they perceive themselves capable of achieving their goals, evaluating priorities and successfully facing the obstacles encountered on their way through a state of pursuit toward their academic goals during the confinement.

The psychological well-being of the students was found at a medium level, with low tendencies. This result points to opportunities for intervention to improve the experience of university students in the development of academic and professional life projects, strengthening self-acceptance and control over what happens in their lives in a context marked by adversity.

The results indicate that bonds should be strengthened in quality and quantity; we believe that bonds were one of the dimensions most affected by the pandemic together with the perceived autonomy to make decisions (Ryff, 1989; Casullo, 2002). In addition to this pandemic situation, other factors related to the critical situation of the country with which students must deal with daily can be incorporated. However, it is at a medium level despite the adversities, which is congruent with the Latin American background described above.

The background described above has emphasized exploring learning in remote emergency education, as well as approaches to mental health as an absence of psychopathology. In this study, we considered psychological well-being as an indicator of mental health related to emotional intelligence (psychic resources) and academic self-efficacy (psychoeducational). It can be stated that the results are congruent with other studies conducted in Venezuela during the COVID-19 pandemic, specifically regarding adequate levels of academic self-efficacy (Romero et al., 2021); however, our results differ from that of the study conducted by Giménez and Medici (2021) regarding emotional intelligence skills. Our results are also congruent with international evidence suggesting that self-efficacy, emotional understanding, and regulation skills may be protective factors for mental health and well-being during a pandemic (Persich et al., 2021; Sabouripour et al., 2021; Sánchez-Ruiz et al., 2021); additionally, in this study, emotional perception established a proportional inverse relationship with psychological well-being, which is congruent with other studies before the pandemic (Extremera and Fernández-Berrocal, 2006; Extremera et al., 2009).

The results confirm that self-efficacy and socioemotional aspects are important for the construction of psychological well-being in university students both pre-pandemic and during it, as they enable the need for competence, autonomy, and relationships according to the self-determination theory (Kohls et al., 2021; Matos Fialho et al., 2021). Similarly, the results are interpreted from Hobfoll’s (1989) conservation of resources theory, which emphasizes that internal resources (such as self-efficacy) at the personal level would allow people to adapt to new, changing, and unfamiliar needs. Before online emergency remote education, the university as an institution could generate external support through either programs or support tutorials that maximized or compensated students’ personal and internal resources that helped to build students’ resilience (Plakhotnik et al., 2021).

The results of this study are congruent with those of an Iranian study, which postulates that self-efficacy is closely linked to optimism, resilience, and the construct of multidimensional psychological well-being of university students during the COVID-19 pandemic. In effect, self-efficacy is a mediator of resilience to each element of well-being: environmental mastery, autonomy, self-acceptance, positive relationships with others, personal growth, and purpose in life. It is suggested that self-efficacy is linked to optimism in the sense of motivating present future-oriented behaviors (Sabouripour et al., 2021). Similarly, emotional stability and self-efficacy were significant predictors of multidimensional psychological well-being in emerging young adults in Nigeria (Bada et al., 2020).

Pre-pandemic scientific literature has also highlighted that emotional intelligence is a personal resource that can be a promoter of both hedonic and eudemonic mental health and psychological well-being (Di Fabio and Kenny, 2016; Sánchez-Álvarez et al., 2020). Similarly, one of the premises put forward by Bandura is that self-efficacy is an active element in the construction and subjectivization of psychological well-being, which can be considered a source of well-being (Sansinenea et al., 2008; Cabanach et al., 2012) even in clinical populations (Suriá and Ortigosa, 2018).

These findings highlight the university educational experience as a space for universal psychoeducational interventions aimed at the promotion of personal resources for both prevention and health promotion, and even from a gender perspective that responds to the diversity of trajectories in the construction of these psychosocial resources (Sawyer et al., 2021). In this study, differences were found only in emotional regulation and the bond dimension of well-being, and mostly among men, evidence suggesting different trajectories according to sex or gender in the experience of the same (Gartzia et al., 2012; García-Álvarez et al., 2020; López et al., 2021). There were significant differences according to age for only psychological well-being in those older than 21 years, which can be explained by the fact that those younger than 21 are in a greater situation of dependence and little autonomy. In addition, age increases the possibility of constructing the elements of multidimensional well-being (Ryff, 2014; Ryff et al., 2021).

We recommend that universities implement social-emotional education strategies that emphasize the development of emotional understanding and regulation, when it comes to factors involved in self-regulated learning and consider academic self-efficacy and its sources of promotion: direct experiences of mastery, vicarious experiences, verbal persuasion, and physiological or emotional activation (Hernández, 2018; Alarcón, 2020) either online or in hybrid face-to-face and online modalities. Although, at the time of writing this article (August 2021), the vaccination process is still ongoing and there are no concrete back-to-school announcements in the country. The objective of these interventions should be to promote health and prevent psychopathology that may jeopardize university permanence of university students, where techniques are provided to develop adequate emotional management and coping strategies to face the different challenges of a university, as well as other complex events (Arias and Giuliani, 2014). We also recommend designing and implementing interventions aimed at promoting agency, a construct formed by self-efficacy, optimism, future orientation, and imagination as a psychological state that allows us to build our well-being and our future (Seligman, 2021). It would be interesting to consider the model healthy university as a management model (Burns et al., 2020).

A limitation of the present study is that the data were collected online due to the current pandemic using non-probability sampling. This represents a bias, given that responses were only obtained from students with Internet access, leaving out those without Internet access. This may have impacted the measurement of the total perception of psychological well-being across all students in a country with marked inequalities. It is also important to note that the participants were limited to Psychology students, the vast majority of whom were female, making a more equitable sample difficult. In this sense, we are recommended that future researchers replicate this study post-pandemic, to compare the results, as well as work with equal groups of men and women to facilitate the analysis of differences by sex and age. However, this was a limitation shared with most of the background described in the introduction on research conducted in this context during the pandemic.

## Data Availability Statement

The raw data supporting the conclusions of this article will be made available by the authors, without undue reservation.

## Ethics Statement

Ethical review and approval was not required for the study on human participants in accordance with the local legislation and institutional requirements. The patients/participants provided their written informed consent to participate in this study.

## Author Contributions

DG-Á contributed to the design of the study, reviewed the literature, interpreted the results, and wrote the manuscript. JH-L contributed to the design of the study, data extraction, data analysis, and review of the abstract and manuscript. RC-R contributed to the literature review, interpretation of the results, and review of the abstract and manuscript. All authors contributed to the manuscript and approved the submitted version.

## Conflict of Interest

The authors declare that the research was conducted in the absence of any commercial or financial relationships that could be construed as a potential conflict of interest.

## Publisher’s Note

All claims expressed in this article are solely those of the authors and do not necessarily represent those of their affiliated organizations, or those of the publisher, the editors and the reviewers. Any product that may be evaluated in this article, or claim that may be made by its manufacturer, is not guaranteed or endorsed by the publisher.
